# Impacts of Inherent Components and Nitrogen Fertilizer on Eating and Cooking Quality of Rice: A Review

**DOI:** 10.3390/foods12132495

**Published:** 2023-06-27

**Authors:** Xiaoqian Guo, Luqi Wang, Guanglong Zhu, Yunji Xu, Tianyao Meng, Weiyang Zhang, Guohui Li, Guisheng Zhou

**Affiliations:** 1 Joint International Laboratory of Agriculture and Agri-Product Safety, Yangzhou University, Yangzhou 225000, Chinag.zhu@yzu.edu.cn (G.Z.);; 2 China-Sudan Joint Laboratory of Crop Salinity and Drought Stress Physiology, The Ministry of Education of China, Yangzhou 225000, China; 3 College of Agriculture, Nanjing Agricultural University, Nanjing 210095, China; 4 Jiangsu Key Laboratory of Crop Cultivation and Physiology, Yangzhou University, Yangzhou 225000, China; 5 Jiangsu Co-Innovation Center for Modern Production Technology of Grain Crops, Yangzhou University, Yangzhou 225000, China; 6 College for Overseas Education, Yangzhou University, Yangzhou 225000, China

**Keywords:** evaluation indicator, amylose, protein, lipid, nitrogen

## Abstract

With the continuous improvement of living standards, the preferences of consumers are shifting to rice varieties with high eating and cooking quality (ECQ). Milled rice is mainly composed of starch, protein, and oil, which constitute the physicochemical basis of rice taste quality. This review summarizes the relationship between rice ECQ and its intrinsic ingredients, and also briefly introduces the effects of nitrogen fertilizer management on rice ECQ. Rice varieties with higher AC usually have more long branches of amylopectin, which leach less when cooking, leading to higher hardness, lower stickinesss, and less panelist preference. High PC impedes starch pasting, and it may be hard for heat and moisture to enter the rice interior, ultimately resulting in worse rice eating quality. Rice with higher lipid content had a brighter luster and better eating quality, and starch lipids in rice have a greater impact on rice eating quality than non-starch lipids. The application of nitrogen fertilizer can enhance rice yield, but it also decreases the ECQ of rice. CRNF has been widely used in cereal crops such as maize, wheat, and rice as a novel, environmentally friendly, and effective fertilizer, and could increase rice quality to a certain extent compared with conventional urea. This review shows a benefit to finding more reasonable nitrogen fertilizer management that can be used to regulate the physical and chemical indicators of rice grains in production and to improve the taste quality of rice without affecting yield.

## 1. Introduction

Rice is one of the principal staple foods that feeds more than 50% of the world’s population [1]. The cultivation, consumption, and market of rice are primarily centered in Asia. It is reported that Asian farmers provided approximately 90% of the total rice production in the world, with China and India jointly contributing almost 51% [2]. According to the International Rice Information System, there are at least 5000 rice varieties that have been released, and many more if traditional rice varieties are included [3]. Rice is cultivated on over 164 Mha of lands in more than 100 countries every year [4]. It is expected that the global demand for rice will grow to 852 million tons by 2035 as the yield per unit area increases year by year [5].

Rice is the major source of nutrition, which can meet the daily need for calorie and protein of over two billion people in the world [6]. Reports showed that rice not only provides primary calories but also is a good source of micronutrients and macronutrients, including proteins, lipids, carbohydrates, dietary fibers, minerals, and vitamins [7,8]. The biological valence of rice protein is 77, ranking first among various cereals and comparable to shrimp (77) and fish (76). Compared with proteins from other plants, rice protein is regarded as an ideal protein source for functional foods due to its hypoallergenic feature and its protein efficiency ratio similar to that of milk casein [9]. In addition, studies showed that rice protein hydrolysates have various biological activities, including antioxidant, antihypertensive, anti-obesity, and anticancer effects [10,11].

Rice is the essential food crop in China, and over 60% of China’s population take rice as their major food [12,13]. From 1980 to 2019, the promotion and utilization of a series of high-yield rice varieties significantly increased both the cultivated area of japonica rice from 2.8 to 9.8 Mha and the yield from 4013.2 to 7429.5 kg ha^−1^ [14]. In the 21st century, the consumption per capita of japonica rice in China has continued to ascend with the annual average consumption increasing from 37.8 to 55.4 kg, leading to a continuous growth in the demand for high-quality japonica rice in the domestic rice market [15].

Rice is the only main cereal which is consumed mainly in whole grain after being cooked [16]. As a main starch source food, after breaking the quantitative bottleneck of rice consumption, the ECQ of rice is the most important character for consumers. Actually, rice quality is a complex feature (Table 1), which is usually difficult to define comprehensively. It can be divided into several main categories, including AQ, MQ, NQ, and ECQ [17]. Rice AQ is influenced by grain shape, transparency, chalkiness, and other indicators. Rice MQ refers to the rice integrity during processing, including the milled rice rate, head rice rate, and roughness rate. Rice NQ reflects the quality and quantity of starch, protein, minerals, vitamins, and other phytochemicals beneficial to human health. Rice ECQ mainly reveals the palatability and properties of cooked rice [18]. Among these, the ECQ is the most essential quality parameter of rice since it meets the satisfaction of customers and controls the price of rice in the market [19].

Rice consumers in varied countries and regions, especially those in countries where rice is the staple food, have intense and often diverse preferences in sensory (taste) characteristics of rice. Generally speaking, the Japanese prefer sticky rice with short grain, which is often utilized to make sushi. In Pakistan, India, and the Middle East, on the contrary, Basmati rice is popular because of its fragrance as well as its elongated and dry grains when cooked [20]. Jasmine-type rice with soft texture and the buttery popcorn-like fragrance is the favorite for many people in Thailand and is served along with Thai-inspired food prepared all over the world [21].

Rice ECQ is a vital factor determining the acceptance of consumers and its economic value in the export markets [22]. In this review, we provide updated information on rice ECQ, including the definition, evaluation indicators, and evaluation methods. Then, we focus on summarizing the research progress on the relationship between rice eating quality and its physicochemical indexes. We also discussed the responses of the ECQ of rice to nitrogen fertilizer.

## 2. Definition, Evaluation Indicators, and Evaluation Methods

### 2.1. Definition and Evaluation Indicators

ECQ refers to the comprehensive estimation of the shape, color, smell, taste, palatability, and sensory characteristics after cooking [23]. To a certain degree, the sensory property of rice, particularly eating quality is a subjective characteristic, varying with social, cultural, and individual factors. Sensory property can be estimated objectively via physicochemical analysis [24]. AC, GT, GC, and the results obtained via a RVA are normally used as indirect indicators to evaluate rice ECQ [25].

A great number of studies have confirmed that the AC is one of the major elements determining the ECQs of rice [26]. It was considered to be positively correlated with the sensory or instrumental values of hardness, and inversely correlated with the stickiness of cooked rice [27]. The rice with excessive AC is typically loose, expansive, and of pale color, and it hardens after cooling and has poor palatability, whereas the rice with low AC is much less expansive, soft, and fluffy, which is well-liked among consumers [28,29]. In light of the rice quality standard, the amylose contents of 18–20% and 18–22% were the optimum for japonica rice and indica rice, respectively, during the variety certification of rice in China [27]. Rice varieties with similar ACs may have different eating qualities. The distinction in eating quality is induced by the starch GT and GC [30,31]. GT and GC are additional indexes that represent starch and the textural characteristics of rice with the same AC [32,33].

GC reflects the hardening degree of cooked rice when it is cooled. It is inversely correlated with amylose [33], and has a correlation with Brabender SBV [31]. Usually, high GC leads to soft cooked rice [27]. According to GC, rice varieties can be classified into three levels: soft (>60 mm, nonflaky, and soft rice), intermediate (41–60 mm, soft but flaky rice), and hard (≤40 mm, hard and very flaky rice) [27].

GT is considered to be the temperature at which starch particles transition from an ordered state to a chaotic state [34,35]. In general, GT of rice varieties can be classified into three levels, including low (55–69 °C), medium (70–74 °C), and high (75–79 °C) [36]. High GT rice is supposed to require longer cooking time, and the texture of cooked rice tends to be much less sticky, particularly when it is cooled [37,38]. GT can be determined indirectly by using a urea or alkaline solution according to the ASV [22]. ASV is negatively correlated with rice GT. Normally, higher ASV leads to low GT and better cooking quality [27]. DSC is another method to measure the GT [22]. DSC determined not only the gelatinization degree of starch but also the ΔH [39].

In addition to AC, the profile features of RVA are used to describe the properties of rice starch due to the fact that its determination simulates the process of heating and cooling of real rice cooking, which can reflect the difference in starch quality between varied rice varieties more sensitively [40]. Several characteristics measured according to the pasting profiles of rice flour throughout the cooking process have been proven to correlate with the eating quality of cooked rice [41,42,43]. Juliano and Pascual found that the PKV was correlated negatively with the stickiness of cooked rice, and positively with its stiffness and consistency [42]. SBV and CSV were negatively correlated with cooked rice stickiness, but positively with each other and the stiffness of cooked rice [44]. Nevertheless, Juliano concluded that PKV could not be considered as an index of the texture of cooked rice [43], and only CSV was correlated with cooked rice stiffness. In addition, it was reported that SBV is useful in predicting the stickiness degree of cooked rice [41]. Rice varieties with high eating quality have features of low SBV, high BDV, and low AC [28,45].

### 2.2. Evaluation Methods

Sensory evaluation and instrumental evaluation are the conventional methods for evaluating rice eating quality, and both of them are studied in cooked rice [46].

The traditional measurement of eating quality mainly depends on human sensory tests. A trained sensory panel will give a sensory profile of cooked rice samples after watching, smelling, and tasting [47]. As shown in Table 2, the sensory test is the essential test of eating quality, which provides information about aroma, appearance, stickiness, hardness, taste, and overall quality [48], but this requires a great quantity of rice samples, and is easily affected by the gender, age, and geographic residential area of the tasters [49,50].

The rice taste analyzer designed in Japan is an accurate, convenient, and efficient tool to evaluate rice cooking and eating characteristics [58]. It converted various physicochemical indexes of the rice into “taste” scores according to the correlations between the preference sensory scores and the near-infrared reflectance measurement values of the key components [58]. Nevertheless, some studies have pointed out that the accuracy of the system needs to be improved [59]. Yang et al. found that the eating quality of rice produced in Jiangsu province was comparable to that of northeastern rice based on the results evaluated by a trained panel. However, taste values obtained from the taste analyzer had different results that northeastern rice was superior to Jiangsu rice [24]. Therefore, the data obtained from the taste analyzer should be interpreted carefully when processing rice samples collected from contrasting growth conditions, and localizing the Japanese taste analyzer through correlating the chemical compositions including moisture, fatty acid, protein, and amylose with the taste scores of Chinese preferences is significant.

## 3. Relationship between Rice ECQ and Its Components

Rice ECQ mainly depends on its intrinsic ingredients [14]. Rice grains consist of about 80–85% starch, 4–10% protein, 1% lipid, and other components with few amounts, and the genetic variation range in biochemical constituents affects rice ECQ [60].

### 3.1. Starch

The proportion of starch in milled rice is about 90% [61]. As the largest component of endosperm of rice grain, starch contains two types of glucose polymers, including amylose and amylopectin. Amylose mostly consists of hundreds of glucose units with linear connections, whereas amylopectin is composed of thousands of glucose units and it is branched to a large degree via the α-1,6-glycosidic bond on the basis of amylose [62]. Rice components varies among varieties, resulting in variability in texture and pasting viscosity [63]. Sanjiva Rao and colleagues were the first to propose that there may be a relation between AC and rice quality [64]. Compared with low AC rice, cooked rice with high AC has slower digestibility and higher nutritional benefits, but worse eating quality [54]. After being cooked, high AC rice tends to have a dry and flaky texture, while rice with low AC would become soft and sticky [24]. Compared with low AC rice, rice with high AC requires less cooking time [65]. Typically, rice is divided into the following groups according to its AC: waxy (0–2%), extremely low (3–9%), low (10–19%), medium (20–25%), and high (>25%) [22]. Feng et al. indicated that too low or too high amylose content had negative effects on rice taste quality [66]. For rice that had more than 20% AC, its texture was hard after cooking, while rice with 15–20% AC had better quality after cooking [67]. Tao et al. also found that rice varieties (IR45427-2B-2-2B-1-1, Vandana, and Topaz) with high AC (≥25.9%) had a higher score for residual particles, roughness, dryness, and hardness, while rice with AC < 20% (Langi, YRF 216, Tachin i, and Hom Mali) is characterized by higher cohesiveness, stickiness, and tooth pack [54]. Medium AC samples (Remant, IR 65598-112-2, and Bengal) scored higher for preference, whereas samples IR45427-2B-2-2B-1-1, Vandana, and Topaz had low eating quality. It is reported that different AC and chain length can influence the formation and preservation of aroma compounds [68]. Kasemwong et al. announced that starch with high AC could complex more aroma molecules [69]. Wulff et al. also found that the association constant between the same aroma compounds and amylose was decided by amylose chain length [70]. An appropriate chain length is conducive to enhance the stability of compounds and the preservation of aroma.

The molecular structure of native rice starch influences the sensory properties of cooked rice [71], such as the remarkable positive correlation between AC of native rice starch and stiffness and the significant negative correlation between AC of native rice starch and stickiness [72]. Nevertheless, the mechanism behind the effects of starch on rice eating quality cannot be fully explained by AC alone. Amylopectin structure has an important impact on the physicochemical attributes of starch [71], which is the major cause for rice quality difference among varieties with similar AC. Rice varieties with higher AC usually have more long branches of amylopectin, which leach less when cooking, leading to higher hardness, lower stickiness, and less panelist preference [54]. Umemoto et al. suggested that the distributions of the short-length chains (DP ≤ 11) of amylopectin and the intermediate-length chains (DP 12–24) in varied rice varieties were different, while the number of long-length chains (DP ≥ 25) was similar [73,74]. The ACR of ΣDP ≤ 11/ΣDP ≤ 24 was seen as the basis for classification according to the actual differences in the chain length as well as chain-length distribution in different rice varieties [75]. All amylopectin varieties can be classified into the following types: type I and type II. The ACR of type I amylopectin was lower than 0.22, while the ACR of type II amylopectin was more than 0.26. The ratio of amylopectin medium chains with DP 13–24 for the hybrid combinations with poor taste values was lower than that for the hybrid combinations with good taste values [76]. However, the ratio of amylopectin short chains with DP 6–10 for the hybrid combinations with poor taste values was larger than that for the hybrid combinations with good taste values. Rice varieties with higher BDV and lower CSV, SBV, CPV, HPV, PaT, and PeT are considered to have higher grain quality, better viscosity, softer texture, and better cold rice texture [75]. Glutinous rice varieties with a lower distribution of intermediate and long chains and a greater distribution of short chains in amylopectin structure brought about lower RVA spectrum characteristic values and GT [77]. Specifically, the distribution of amylopectin short chains with DP 6–11 in glutinous rice varieties had remarkable and negative correlation with CSV, CPV, HPV, PKV, PeT, and PaT. The distribution of intermediate-long chains ranging from DP 12 to 24 was significantly positively correlated with CSV, CPV, HPV, PaT, and PeT. Amylopectin long chains with DP 25–36 also had significant and negative correlation with PaT. Previous studies found that chemically isolated rice amylopectin produced three kinds of anhydro glucose chain populations (long B, medium-B, and A plus short B) upon debranching [78,79,80]. In most of research mentioned above, the stickiness had negative correlation with the stiffness or firmness. Generally, stickiness is attributed to amylopectin, particularly the short chains of amylopectin (A and short B chains) [80,81,82]. The increase in the proportion of amylopectin short chains produces greater opportunities for bonding and molecular interaction; hence, more force is needed to separate the granules, eventually resulting in higher stickiness [16]. The decreased amylopectin medium chains (DP 13–24) as well as the higher number of short chains (DP 6–12) will lead to more amylopectin leaching during RVA measurement, therefore causing higher viscosity [83]. Hybrid rice with more amylopectin long chains has a harder texture when cooked [76]. The larger proportions of amylopectin long chains inhibit the swelling and gelatinization of starch particles, bringing about poor taste values of hybrid rice. Amylose-lipid compounds can form an insoluble layer around the particles to prevent the entry of water [84]. The long amylopectin chains interact with other ingredients in rice grains, including lipids, proteins, and non-starch polysaccharides, leading to a harder texture and restricting the swelling of starch [82,85]. In addition, a longer lipid chain length as well as higher concentration leads to delayed pasting and gelatinization of starch suspension, leading to a firmer cooked rice texture, whereas a short chain has the reverse effect [86]. Moreover, Peng et al. reported, for the first time, that the larger diversity of sizes and forms of starch granules might affect hybrid rice eating quality [76]. Larger native starch particles would have much more bonding points with water, which benefits the uptake of more water during cooking, and thus reducing the hardness [87].

The biosynthesis of rice starch involves numerous enzymes, including GBSSI, SSS, AGPase, DBE, and SBE [88,89,90]. Each enzyme is encoded by a corresponding gene. To date, more than 20 genes associated with starch biosynthesis have been found, including genes that encode AGPase subunits *AGPlar*, *AGPis*, *AGPsma*; multiple genes generated by different splicing modes, genes that encoding starch synthase, including *GBSSII*, *SSI*, *SSIIa*, *SSIIb*, *SSIIc*, *SSIIIa*, *SSIIIb*, *SSIVa*, *SSIVb*, and *Wx*; genes that encode debranching enzymes *ISA*, *PUL*; and genes that encode starch branching enzymes *SBE1*, *SBE3*, *SBE4* [91,92,93]. The *Wx* gene is the major decisive factor of AC and performs a determining role in rice ECQ [26]. So far, a series of *Wx* alleles have been reported, such as *Wx*, *Wx^lv^*, *Wx^in^*, *Wx^a^*, *Wx^b^*, *Wx^mw/la^*, *Wx^op/hp^*, *Wx^mp^*, and *Wx^mq^* [94,95,96,97,98]. In normal rice varieties, high AC level (>25%) are decided by the *Wx^lv^* and *Wx^a^* alleles; nevertheless, the *Wx^lv^* allele was recognized as the ancestor type of the *Wx* gene, conducing to a higher AC level than that of the *Wx^a^* allele [99]. With regard to other *Wx* alleles, *Wx^mp^*, *Wx^mq^*, and *Wx^op/hp^* conduce to a very low AC level (8–12%); *Wx^mw/la^* results in a low AC (about 14%); *Wx^b^* also leads to a low AC (about 15%); *Wx^in^* contributes to a medium AC (about 20%) [94,97,98,100]. Feng et al. also confirmed that rice carrying *Wx^lv^* allele tends to have larger AC than rice carrying other *Wx* alleles [99]. The allele *Wx^b^* (usually exists in japonica cultivars) is a low-amylose allele along with soft texture [101]. The *Wx^a^* allele (mainly distributed in indica cultivars) has a higher AC as well as a firmer cooked rice texture. The AC of *Wx^a^* was significantly higher than that of *Wx^b^*. In terms of RVA parameters, SBV, CPV, and HPV were higher in rice carrying *Wx^a^* than those in rice with *Wx^b^*, while BDV and PKV were lower in rice with allele *Wx^a^* than those in rice carrying *Wx^b^* [102]. In regards to the GT, the values of Tc, Tp, and To of the rice with *Wx^a^* allele were slightly lower than those carrying the *Wx^b^* allele, which suggested that *Wx* was at least a secondary factor affecting the GT. Compared with rice with the *Wx^a^* and *Wx^b^* alleles, rice carrying the allele *Wx^lv^* had higher AC but softer GC [99]. Starch synthase gene *SSIIa* is the major gene controlling rice GT, and Umemoto et al. located the *SSIIa* gene at the *ALK* site of the short arm of rice chromosome 6 [74]. The alleles *SSIIa* and *SSIIIa* derived from a non-semi waxy parent showed a trend of increasing AC. *SSIIa^wj^* allele could enhance the BDV, CPV, HPV, and PKV, while reducing the SBV and CSV. In regard to the *SSIIIa^wj^* allele, the opposite was true [26]. Luo et al. proved that in the inbred line population of Nip and IR64, the *SSI^j^* allele engendered fewer short amylopectin chains (DP 8–12) than the *SSI^i^* allele [103]. *Wx* and *SSIIa* genes have great interaction effects on starch physicochemical characteristics. Huang et al. demonstrated that in transgenic rice, the expression of *Wx* was declined with *SSIIa* expression inhibited by RNAi, suggesting that there was an interaction between *SSIIa* and *Wx* expression [104]. When cooked rice was warm, the taste value of grains from Nip (*Wx^b^*/*SSI^j^*) was significantly higher than Nip (*Wx^b^*/*SSI^i^*), and gains from waxy rice had a similar taste value [105]. In addition, *SSIV-2*, *ISA*, and *SBE3* are secondary genes that influenced GT additively [76].

### 3.2. Protein

Although there is a positive correlation between amylose content and cooked rice hardness, the physical characteristics of Chinese cooked rice are primarily affected by its PC. The structure and content of starch decide the overall stiffness of cooked rice, while protein determines rice surface hardness [106]. Protein is the second most significant ingredient of rice after starch, with a content of about 40–180 g kg^−1^ [107,108]. Grain PC varies depending on rice varieties, ranging from 5.9% to 16.5% in japonica rice and from 4.9% to 19.3% in indica rice [109]. Rice PC is one of the most vital factors affecting ECQ. Although the PC of rice determines the nutritional quality, it also has a negative impact on cooking features by affecting the cohesiveness and hardness of grain. Low-PC rice usually has better eating quality [110,111,112,113]. Previous studies implied that higher PC seriously decreased the rice taste value, which might be due to the fact that PC is highly negatively correlated with BDV and PKV and positively correlated with hardness [14]. When grain PC surpasses 7%, the ECQ of rice tends to decline [114,115]. Li et al. also found that the japonica rice with the highest taste value had the lowest AC, SBV, and PC [116]. Proteins themselves have no flavor in rice substrate, but amino acids, the base unit of protein, can provide abundant precursors for rice aroma formation. The volatile sulfur-containing compounds generated by protein oxidation or decomposition during heating, such as dimethyl sulfide and hydrogen sulfide, can also influence rice aroma [117]. Moreover, Yanova et al. reported that PC directly influences the hygroscopicity of rice grains [118]. High PC, few gaps between starch grains, compact structure of rice grains, less water absorption, and slow water absorption lead to longer cooking time, insufficient starch gelatinization, lower viscosity, and looser texture of rice [119]. Protein contends with starch for water during the cooking process of rice [120,121], and limits the swelling of rice starch granules through forming disulfide-bond networks [112,122]. Meanwhile, the denatured protein forms a gel matrix after heating, which strengthens starch integrity, thus decreasing the viscosity and enhancing the hardness of cooked rice [123]. Asimi et al. evaluated 12 newly bred rice varieties and found that the hardness of H2-73 was significantly higher than other rice samples due to its high PC and AC [119]. The PC in each layer of low eating quality rice was higher than that of high eating quality rice, particularly in the outer layer [124]. Rice outer layer was the main channel for water to enter the rice interior, and excessive PC in the rice outer layer may inhibit water absorption of rice [125]. Additionally, high PC impedes starch pasting [126]. Hence, it may be hard for heat and moisture to enter the rice interior, ultimately resulting in worse rice eating quality.

SSPs constitute a large proportion of grain proteins in rice [127]. There are four types of rice grain SSPs with different solubility-related physical characteristics: prolamin, albumin, glutelin, and globulin. Each of these protein fractions exhibit different levels of aqueous solubility, thus it is possible that they affect the quality of rice by having an influence on starch hydration rate during cooking [107]. The content, distribution, and features of the four types of SSPs in rice are quite different, leading to different effects on rice eating quality [128]. Globulin and albumin are distributed at the outer regions of rice endosperm, while glutelin are stored towards the center. On the contrary, prolamin is distributed evenly in the whole rice endosperm [129]. Glutelin is abundant in essential amino acids and is the richest SSP, accounting for about 80% of total SSPs. Zhang et al. found that the contents of glutelin and prolamin were lower but albumin content was higher in varieties with higher eating quality [130]. Rice prolamin and glutelin contents could enhance or decline together, and the content of albumin is significantly negatively correlated with AC [131,132]. Compared with the medium-AC varieties, the low-AC varieties usually have a higher albumin content. These results indicated that decreased prolamin and glutelin contents and enhanced albumin content could greatly improve the ECQ of rice despite rice glutelin being widely considered to have higher nutritional value in contrast to other storage proteins [88,130]. Balindong et al. found a significant correlation between eating quality and protein compositions of rice with two grain shapes, and reported that the total content of prolamin and the ratio of prolamin/(glutelin + prolamin) were positively correlated with pasting properties [107]. However, they also indicated that globulin exhibited very low levels of variation between and within different kinds of rice, which reflected in their little to no correlation with the texture analyzer or RVA parameters. Globulins are synthesized at the early stage of rice grain development and may function as structural proteins to maintain the integrity of protein body [133,134], and this may be the reason responsible for their low level of variation.

Protein in rice gains can interact with starch to form a network structure, resulting in increased starch solubility and swelling, decreased TV and PKV, enhanced SBV and PaT, containment of starch gelatinization, and declined viscosity of rice after cooking [134]. The generation of disulfide bonds enhanced the level of cross-linking between starch and protein, allowing the protein to form strong networks around the starch, restricting starch expansion [135]. When observing the interaction between starch and glutelin, the hydrophobic amino acids and hydrophilic groups in glutelin adhere to the surface of the starch granule through hydrophobic interactions or hydrogen bonds [136]. This interaction could affect the structure of starch granules and influence its gelatinization. The higher prolamin content is related to higher BDV but lower gumminess, adhesiveness, and stiffness of the gel generated from the flour, whereas albumin is positively related to gel hardness, pasting viscosity, BDV, and FV, but negatively linked with gel adhesiveness [137,138]. Moreover, coordinating the balance between protein and amylose is the key to enhance rice eating quality. Yan et al. revealed that when the AC in conventional japonica rice varied from 13.9% to 19.4%, there was a remarkable negative correlation between PC and eating quality [139]. Liu et al. reported that when AC changed in the narrow range, for example, 7.35–12.50% or 14.11–19.98%, eating properties had no significant correlation with AC, and instead they were significantly correlated with PC [140]. This suggested that eating characteristics are mainly affected by PC when AC varies within a narrow range. When PC varied in wide range (6.04–9.32%), it was significantly correlated with eating properties. However, when PC spanned in a range (7.86–9.32%), the impact of PC on eating quality was not significant. These studies indicate that the variation ranges of AC and PC may affect the relationships among PC, AC, and eating quality. In addition, Liu et al. also found that in order to improve rice eating quality (taste value > 60), the PC in high AC type varieties (14.11–19.98%) should be lower than 6.98%, and the AC in high PC type varieties (7.86–9.32%) should be less than 12.67% [140].

### 3.3. Lipid

Lipid is one of the three major essential nutritional ingredients in rice grains, with milled and brown rice having 0.8% and 3% lipid contents, respectively [141,142]. Although lipid content is low in rice, it is important in energy storage, cell membrane components, and signal transduction [143,144]. Rice lipids are mainly distributed in the embryo, with a content of 34.1–36.5%, followed by aleurone layer and endosperm, with a lipid content of 19.4–25.5% and 0.41–0.81%, respectively [145]. Lipid is a vital nutrient that surrounds starch particles in rice and can interact with amylose during starch gelatinization. It is usually bound with amylose and amylopectin to form complexes, and thus, affect GC and the viscosity of rice elasticity and texture [146]. Rice with higher lipid content had a brighter luster and better eating quality [147]. The role of rice lipids was studied in the variety Koshihikari planted in two places, wherein the removal of lipids was found to enhance the firmness of cooked rice [148]. Fitzgerald et al. investigated the effect of rice lipids on RVA characteristics, and found that the main role of lipids in RVA properties in rice was to change PKV and FV, possibly due to the release of more amylose through the loss of amylose-lipid compounds upon degreasing [149]. Moreover, the combination of protein and lipid peroxide formed by the automatic oxidation of UFAs reduces the solubility of protein [150,151]. In the lipid system, lipid can affect the release and volatilization of aroma by reducing the vapor pressure of aroma compounds, hence changing the release time and headspace concentration. Bi et al. found that the addition of edible oil in cooked rice weakened aroma release during consumption, whereas the taste of rice was more mellow, possibly because lipids retained more aroma compounds in cooked rice [152].

Lipids are usually be divided into two categories, non-starch lipids and starch lipids (bound lipids), according to whether they bound with starch or not. Non-starch lipids including glycerophospholipids (phosphatidylinositol, phosphatidylethanolamine, phosphatidylcholine, and their lyso form) and glycerolipids (triglyceride and diglyceride) are mainly present in the oil bodies of embryo and the aleurone layer, while starch lipids bound with starch particles exist in the rice endosperm [153,154]. Starch lipids, mainly composed of free FA and lysophospholipid, play a vital role in the biosynthesis of starch and thereby affect rice ECQ [145]. Previous studies have indicated that compared to starch and protein contents of rice grain, the lipid content, particularly the starch lipid content of milled rice, had a remarkable impact on rice ECQ, which enhances with the increase in the starch lipid content of milled rice [155,156]. Studies also reported that starch lipids in rice have a greater impact on rice eating quality than non-starch lipids [157]. Starch lipids complexes, formed during heating–cooling process or in natural starches, decreased starch solubility, and gelatinization [158,159]. Lipids and amylose compounds formed during cooking can entangle with amylopectin molecules and limit the swelling of particles, leading to incomplete gelatinization during cooking, which causes a lower PKV and a higher PaT [160,161]. Zhang et al. observed a significant enhancement in pasting viscosity via removing both starch lipids and non-starch lipids instead of taking out non-starch lipid only [162]. In addition, the presence of rice starch lipids can impede the contact of starch particles with digestive enzymes, and thereby decrease the digestibility of starch particles [126].

Lipids are resolved by lipase into FA and glycerin. Most of the FA ingredients are high-quality UFAs, accounting for about 75% of rice total FAs. They not only have great nutritional value, but also are closely related to the ECQ of rice [34,153,163,164]. It was reported that lipids content, particularly the content of unsaturated lipid, is positively correlated with the fragrance and overall quality of rice [165,166]. Increasing UFAs content can significantly enhance rice eating quality.

## 4. Nitrogen Fertilizer Affecting Rice Eating Quality

In addition to intrinsic ingredients, the eating quality of rice is also greatly affected by nitrogen fertilizer (Table 3). In general, as a vital cultivation measure, the application of nitrogen fertilizer can enhance rice yield, but it decreases the ECQ of rice. Many studies showed that the contents of protein and amino acid in rice is enhanced with nitrogen application rate, whereas starch and amylose contents are reduced accordingly [113,167,168]. Jiang et al. demonstrated that panicle nitrogen fertilizer degraded the ECQ of inferior and superior grains of japonica rice, which enhanced the hardness, reduced taste value, and deteriorated pasting properties. This was associated with the fact that panicle nitrogen fertilizer improved PC and declined starch and crude fat content [169]. Through a two-year experiment, Zhao et al. found that excessive nitrogen decreased the average and maximum grain filling rate of inferior grains and superior grains of japonica rice and lengthened the active period of grain filling, and deteriorated the ECQ, suggesting that excessive nitrogen causes the lower rice grain quality by inhibiting grain filling. Compared with superior grains, the quality parameters of inferior grains were more sensitive to nitrogen fertilizer [170]. However, some studies proved that the responses of rice starch to nitrogen fertilizer varied among varieties. Hu et al. investigated the responses of the quality of two rice varieties to nitrogen application. They found that the BDV and PKV of giant embryo rice (J20) reduced and its PaT and SBV improved with increasing nitrogen, whereas Koshihikari had an opposite trend, but the contents of protein and protein component of both varieties were enhanced with increasing nitrogen, with gliadin being the most sensitive protein ingredient to nitrogen fertilizer [171]. Therefore, the nitrogen fertilizer treatment suitable for different rice varieties should not be the same. Developing cultivation measures based on specific varieties and needs can greatly meet the needs of high yield and quality in rice production.

The changes in starch structure and protein distribution significantly affect rice eating quality. With the enhancement of nitrogen rate, the mean diameter of starch volume was lower; the average chain length of amylopectin was longer; and the starch relative crystallinity was higher. The above changes in starch structure caused increased starch solubility, expansion power, and gelatinization enthalpy, and resulted in reduced pasting viscosity, retrogradation enthalpy, and retrogradation percentage, thereby contributing to enhanced stickiness and hardness of rice and decreased taste value [169]. Nitrogen fertilizer largely improved PC in rice outer layer, and the increase in the outer layer was significantly larger than those in rice middle and inner layer with increasing nitrogen rates [125]. During cooking, the rice outer layer directly contacts water. More protein content in the rice outer layer could restrain water absorption, which may make the rice outer layer difficult to gelatinize, which eventually affects rice eating quality. Additionally, high nitrogen rate causes more protein to stick around the amyloplasts, shaping a honeycomb structure, and this suppresses starch gelatinization during cooking [172]. Moreover, under high nitrogen rate, the protein in rice has more β-sheets, which can retard the rate of water entering into the starch interior and avoid the devastation of short-range ordered structure of starch.

Although nitrogen application decreases rice eating quality, some types of nitrogen fertilizers can increase the quality to a certain extent compared with conventional urea. In recent years, CRNF has been widely used in cereal crops such as maize, wheat, and rice as a novel, environmentally friendly, and effective fertilizer [174]. It can slowly release nitrogen into the soil throughout various growth periods to guarantee a continuous supply of nitrogen during crop growth [175]. Zhao et al. investigated the effects of the combined application of normal urea and controlled release urea (at different nitrogen ratios) on the yield, grain quality, and nitrogen efficiency of rice. They obtained a lower hardness of cooked rice treated with controlled release urea compared with that treated with fractionated urea [176]. It was reported by Wei et al. that the CPV, HPV, BDV, PKV, and CSV of rice under the treatments of slow or controlled release fertilizer were higher than those under common urea; and GC, CPV, HPV, and PKV were all positively correlated with viscosity, taste value, and appearance, while BDV and PKV were negatively related to hardness of cooked rice [177]. Xu et al. also found that milled rice starch content, MQ, AQ, as well as the taste value of rice treatment with CRNF were significantly larger than conventional urea, while glutenin, gliadin, and protein contents and hardness of rice had the reverse trends [178]. The higher PC in rice treated with conventional urea led to higher GT and firmer rice, which may be the reason that rice ECQ in the conventional urea was lower than that in the CRNF treatment. Liu et al. demonstrated that a controlled release fertilizer treatment produced higher PC in rice than no fertilizer treatment, but differently to the above studies, they observed that rice treated with controlled release fertilizer had lower rice AC compared with no and conventional fertilizer [179]. The relative improvement of nitrogen metabolism in a single rice cultivar with varied fertilizer treatments tends to inhibit the carbon metabolism during grain filling [180]. Thereby, slow or controlled release fertilizer or fertilization mode with continuous release of nitrogen, especially at the late growth stage of rice, had a tendency to enhance protein content while reducing amylopectin, amylose, and starch contents [177]. Adopting appropriate fertilizer types and fertilizer operations can improve rice ECQ while ensuring high yield, which can meet the goal of highly efficient and high-quality rice production.

## 5. Conclusions and Future Perspectives

The quality of rice is crucial for its commercial value. With the improvement of people’s living standards, new requirements have been put forward for rice ECQ. High quality rice varieties need to have good ECQ on the premise of high yield. For a long time, rice production has focused on increasing yield, and the research on the rice quality has started relatively late, especially in terms of ECQ. Among the multiple indicators used to measure rice quality, ECQ is the most complex. Generally, artificial taste or indirect traits such as AC, PC, and RVA values are used to evaluate rice ECQ. Starch is the most important component in rice, accounting for about 90% of milled rice. Therefore, the content, structure, and distribution of starch are closely related to rice ECQ. Protein is the second most abundant component in rice, and more and more studies have shown that protein has a significant impact on grain water absorption and starch gelatinization during the cooking process. Lipid is one of the three basic nutritional components in rice. Although lipid content in rice is low, it significantly regulates the elasticity, viscosity, and texture of rice. Hence, in evaluating rice ECQ, one cannot solely rely on a single indicator or method. We should start from the physical and chemical foundation of rice itself, combine the results from artificial taste and various testing instruments, and consider the preferences of people in different regions to evaluate the ECQ of rice more scientifically and systematically.

Rice ECQ is mainly influenced by factors such as genetics, environmental factors, and cultivation measures. Nitrogen application is an important agronomic measure in rice production and has an impact on the formation of rice quality, especially when it is applied after the heading stage. Further exploration of the responses of rice physicochemical indicators and ECQ to nitrogen fertilizer is of great significance for revealing the physicochemical mechanisms underlying the formation of rice ECQ and the mechanisms by which nitrogen regulates rice ECQ. Recent studies have shown that rice yield and ECQ are extremely sensitive to different nitrogen regulation practices. Nitrogen fertilizer affects the growth and development of rice and regulates rice physiological and biochemical metabolism, which not only affects the content of various physical and chemical substances in mature grains, but also has a significant impact on the structure and distribution of each physical and chemical substance in the grains. It is generally believed that the application of nitrogen fertilizer can reduce rice ECQ, but the physiological mechanisms of quality deterioration in different rice varieties after nitrogen application may not be the same. This provides us with a new idea that the combination of suitable varieties and matching fertilizers may not significantly affect rice ECQ while increasing yield. Therefore, in the future work of promoting varieties and popularizing cultivation measures, matching varieties and fertilizer management as a whole is a new strategy to achieve both high yield and good ECQ in rice production.

## Figures and Tables

**Table 1:** The classification of rice quality.

Rice Quality	Property	Reference
Appearance	Shape, transparency, chalkiness	Lu et al. [17] Li et al. [18]
Milling	Milled rice rate, head rice rate, roughness rate	
Cooking and eating	Amylose content, gel consistency, gelatinization temperature	
Nutritional	Starch, protein, lipid, micronutrient	

**Table 2:** An overview of literature data on the sensory analysis of cooked rice.

Reference	Rice Varieties	Tasters	Sensory Characteristics	Overall Quality
Lee et al. [47]	24 rice cultivars	14 trained members	–	Samkwang was the best EQ cultivar, followed by Ilpum, Gopum, Koshihikari, and Cheongpum. Te least favored cultivars were Namil, followed by Samnam and Palgong.
Buenafe et al. [51]	5 classes of the benchmark varieties	5 males and females from India	Appearance, elongation, tenderness on touching, cohesiveness, tenderness on chewing, taste, and aroma.	Classes A and E were deemed the most acceptable varieties with an overall acceptability of 3.9 and 4.0, respectively, out of 5.
Mane et al. [52]	LsiBFf, LWF, LBFf, IBFf, IWF	120 consumers	Grain color, grain size, broken grains, whole grains, pasty texture, heterogeneous size, medium grains, easy to digest, hard texture, good taste, fine grains, too many impurities, too small grains, soft texture, fragrant, white color, overcooked, sticky texture, well-cooked, old aftertaste, scattered, typical rice odor, clean, good for ceeb, beautiful, and dirty white.	(LWF and IWF) > LBFf > IBFf > LsiBFf
Mao et al. [53]	RILs (n = 101) and BILs (n = 48) constructed using cv. Toyonishiki (TSI)/cv. Qishanzhan (QSZ) and TSI/QSZ//TSI	20 panelists	Odor, appearance structure, palatability, taste, and texture of cold rice.	TSI > BILs > RILs > QSZ
Tao et al. [54]	11 rice cultivars	5 males and 5 females, all from China and all resident in Brisbane, Australia, for at least one year	Stickiness, roughness, hardness, cohesiveness, toothpack, dryness, and residual rice.	Hom Mali, IR 65598-112-2, and Bengal scored higher for preference, whereas IR45427-2B-2-2B-1-1, Vandana and Topaz had low scores of eating quality.
Park et al. [55]	Hwayeong, and Wandoaengmi6, were crossed to develop a recombinant inbred lines (RILs) population	adult 7–15 panelists	Appearance, glossiness, aroma, stickiness, eating taste, hardness, and overall eating quality.	Wandoaengmi6 was the best.
Han et al. [56]	Bengal, Cypress, Cocodrie, and Francis	7 trained panelists	Manual stickiness, initial cohesion, adhesion to lips, hardness, cohesiveness, cohesiveness of mass, roughness of mass, toothpull, number of chews, residual film, and toothpack.	–
Hu et al. [57]	Zhongzheyou 8	–	Color, luster, grain integrity, viscosity, elasticity, hardness, taste, and cold rice texture.	Rice edible quality had significant decrease after being stored two years.

**Table 3:** Impacts of nitrogen fertilizer on rice ECQ.

Reference	Rice Varieties	N Rate (kg N ha^−1^)	Taste Value and Texture	Intrinsic Component Content	Intrinsic Component Structure and Distribution	Physical Properties
Zhou et al. [113]	Shendao 47 Jingyou 586	0 140 180 220	–	PC increased, AC decreased.	–	GC decreased. PKV, TV, FV, BDV decreased. SBV increased. Rice starch had a higher PaT and a longer PeT under a high N rate for Jingyou 586.
Shi et al. [125]	Huanghuazhan	0 50 100 350	Taste value decreased, hardness increased, stickness not changed.	PC increased, AC decreased.	PC in rice outer layer increased and the short-range ordered structure of starch decreased. The relative crystallinity of starch and the proportion of large granular starch first decreased and then increased.	PKV and FV decreased, BDV first decreased and then increased, SBV first increased and then decreased.
Xiong et al. [167]	Yangdao 6	0 (N0) 8 g pot^−1^ (N1)	–	PC increased, AC decreased.	N improved the distributing of amyloplasts. With N0 treatment, most amyloplasts were elliptic and their arrangements were loosen especially in the ventral side. Conversely, most amyloplasts with N showed a shape of crystal and their arrangements were more compact. N improved the size, quantities and distribution of proteinoplasts in rice.	–
Singh et al. [168]	Punjab Mehak 1 Pusa Basmati 1121 Punjab Basmati 2	0 20 40 60	Hardness, cohesiveness, and chewiness increased while adhesiveness decreased.	PC and ash content increased, AC decreased.	–	Cooking time and cooked grain length/breadth ratio of Punjab Mehak 1 and Pusa Basmati 1121 increased. Gelatinization transition temperatures of Pusa Basmati 1121 and Punjab Mehak 1 starch decreased, while that of Punjab Basmati 2 increased slightly. The ΔH of the starches from all the rice cultivar decreased.
Jiang et al. [169]	Nanjing 9108 Nanjing 0212	0 60 120 180	Taste value of superior and inferior grains of japonica rice reduced, while hardness and stickiness increased.	The content of total starch, amylose, amylopectin and the ratio of amylose/amylopectin decreased. The crude fat content decreased but had an insensitive response to the gradient of N. Total protein and protein component contents increased. The increase in total protein content by PNF was mainly related to prolamin and glutenin.	The proportion of small and medium starch granules increased, while the proportion of large starch granules decreased. The starch volume mean diameter decreased. A chain content decreased, and B2 chain and B3 chain contents increased, the average chain length of amylopectin increased and (A + B1)/(B2 + B3) decreased. Starch relative crystallinity increased. The starch amorphous region and single helix content decreased, and the double helix content increased. Starch solubility and swelling power improved.	The PKV, hot viscosity, and FV decreased. The SBV decreased in the beginning and then increased, and the PaT increased, while the BDV showed few differences. The ΔH increased, and the retrogradation enthalpy and percentage decreased.
Zhao et al. [170]	Nanjing 9108 Ningjing 7	0 195 270 345	Taste value decreased, hardness increased.	AC decreased, protein component content increased.	–	The viscosity, balance, and gel consistency decreased.
Hu et al. [171]	J20 Koshihikari	0 (N0) 90 (N1) 135 (N2) 180 (N3) 225 (N4)	–	PC and protein components contents increased, and the albumin contents under the N4 treatment were significantly different from those under the N0 treatment.	–	–
Shi et al. [172]	Huanghuazhan	0 (N0) 350 (N1)	Hardness increased, while exterior, taste, and taste value decreased.	PC increased, AC decreased.	More protein bodies surrounding the amyloplasts. Compared with N0, rice at N1 at 0 min had more large granular matter, and there was some granular matter around the large amyloplasts. Rice protein had more β-sheets.	The PKV and BDV decreased. After cooking for 10 min, almost all amyloplast were converted into starch paste at N0, but some amyloplast was still structurally intact at N1. As the cooking time increased, the starch began to gelatinize, and more amyloplasts became flat and smooth. At N1, it still showed more considerable flat material.
Liu et al. [173]	Xiushui 134	0 (N0) 120 (N1) 165 (N2) 210 (N3)	–	The contents of total starch, amylopectin and amylose decreased at N3, while N1 and N2 had no significant effects on these three parameters. PC increased. Except for cysteine, isoleucine and proline content, the other 14 amino acid contents significantly increased.	–	–
Liang et al. [128]	Yanfeng 47	160 (N1) 210 (N2) 260 (N3) 315 (N4) 420 (N5)	Taste values declined with increasing N rates. N rate had little impact on cohesiveness and adhesiveness. Compared with N3, N2 increased springiness, whereas N5 increased hardness, chewiness, and springiness.	N rates affected glutelin and globulin content. Met content increase observed in globulin with increasing N rate. N5 reduced amino acids contents except Cys, Met, Ile and Tyr in albumin. Compared to N1, N5 increased Ser, Glu, Ala, Ile, Leu, Tyr, Phe, and Pro contents in prolamin and decreased sulfur-containing amino acids (Cys and Met). N rate did not affect the amino acid composition of glutelin.	Nitrogen treatment had no significant influence on the secondary structure of glutelin, globulin, and albumin. Compared with N1, N4, and N5 significantly decreased the ratio of α-helix of prolamin.	With the increasing N rate, PKV, TV, and FV first increased and then decreased, and the peak value appeared at N2. Compared with N1, N4 and N5 reduced the viscosity index. With increased N rate, rice swelling rate and softness increased at first and then decreased, achieving the highest point at N2. N3 and N4 reduced PaT compared with N1.

AC: amylose content, PC: protein content, GC: gel consistency, PKV: peak viscosity, TV: trough viscosity, FV: final viscosity, BDV: breakdown viscosity, SBV: setback viscosity, PaT: pasting temperature, PeT: peak time, ΔH: gelatinization enthalpy, Met: methionine, Cys: cysteine, Ile: isoleucine, Tyr: tyrosine, Ser: serine, Glu: glutamic acid, Ala: alanine, Leu: leucine, Phe: phenylalanine, Pro: proline.

## Data Availability

No new data were created or analyzed in this study. Data sharing is not applicable to this article.

## Abbreviations

AC: amylose content; ACR: amylopectin chain ratio; AGPase: ADPGlc pyrophosphorylase; AQ: appearance quality; ASV: alkali spreading value; BDV: breakdown viscosity; CPV: cool paste viscosity; CRNF: controlled release nitrogen fertilizer; CSV: consistency viscosity; DBE: starch debranching enzyme; DSC: differential scanning calorimetry; ECQ: eating and cooking quality; FA: fatty acid; FV: final viscosity; GBSSI: granule bound starch synthase I; GC: gel consistency; GT: gelatinization temperature; HPV: hot paste viscosity; MQ: milling quality; Nip: Nipponbare; NQ: nutritional quality; PaT: pasting temperature; PC: protein content; PeT: peak time; PKV: peak viscosity; RVA: rapid viscosity analyzer; SBE: starch branching enzyme; SBV: setback viscosity; SSP: seed storage protein; SSS: soluble starch synthase; Tc: conclusion temperature; To: onset temperature; Tp: peak temperature; TV: trough viscosity; UFA: unsaturated fatty acid; ΔH: gelatinization enthalpy.
